# Plasmodium falciparum CRK5 Is Critical for Male Gametogenesis and Infection of the Mosquito

**DOI:** 10.1128/mbio.02227-22

**Published:** 2022-09-26

**Authors:** Sudhir Kumar, Olivia R. Gargaro, Stefan H. I. Kappe

**Affiliations:** a Center for Global Infectious Disease Research, Seattle Children’s Research Institute, Seattle, Washington, USA; b Department of Pediatrics, University of Washington, Seattle, Washington, USA; c Department of Global Health, University of Washington, Seattle, Washington, USA; Albert Einstein College of Medicine

**Keywords:** CRK5, gametocyte, exflagellation, mosquito, transmission

## Abstract

Cyclin-dependent kinases (CDKs) and cyclins are critical cell cycle regulators in eukaryotes. In this study, we functionally characterized a CDK-related kinase (CRK5) of the human malaria parasite Plasmodium falciparum. P. falciparum CRK5 (*Pf*CRK5) was expressed in asexual blood stages and sexual gametocyte stages, but showed male gametocyte- specific expression. In contrast to previous findings, we showed that gene deletion *Pfcrk5^−^* parasites grew normally as asexual stages and underwent normal gametocytogenesis to stage V gametocytes. However, *Pfcrk5^−^* parasites showed a severe defect in male gametogenesis, which was evident by a significant reduction in the emergence of male gametes (exflagellation). This defect caused a severe reduction of parasite transmission to the mosquito. Genetic crosses performed using sex-specific sterile transgenic parasites revealed that *Pfcrk5^−^* parasites suffered a defect in male fertility but female gametes were fertile. Taken together, these results demonstrate that *Pf*CRK5 is a critical sexual stage kinase which regulates male gametogenesis and transmission to the mosquito.

## INTRODUCTION

Plasmodium falciparum remains the main causative agent of malaria, a disease with significant mortality and morbidity in developing countries across the world. P. falciparum is an obligate intracellular parasite with its life cycle alternating between a human host and Anopheline mosquitoes. Inside the red blood cells of a human host, the parasite cyclically replicates asexually over ~48 h periods, undergoing development as rings, trophozoites, and schizonts, ultimately forming new infectious merozoites. Some of the asexually replicating parasites commit and differentiate into sexual stages called gametocytes and develop through a number of morphologically distinct stages (stage I-V) over a 2-week period. Stage V gametocytes are taken up by the mosquito in blood meal where they rapidly get activated to form gametes (female; macrogametes and male; microgametes). A male gametocyte forms 8 flagellar microgametes, while a female gametocyte forms a single macrogamete (1, 2). These gametes fuse to form a short-lived zygote, which transforms into a motile ookinete. These stages penetrate the mosquito midgut epithelium and further develop as oocysts and eventually produce transmissible sporozoites over a 2-week period.

Factors controlling gametogenesis include increase in pH (1), a drop in temperature (1, 2), and/or exposure to xanthurenic acid (XA), a metabolite of tryptophan (3, 4). Gametogenesis is further linked to mobilization of intracellular Calcium (Ca^2+^) via protein kinase G (PKG) (5), and Calcium-dependent protein kinases (CDPKs), CDPK1 (6), CDPK2 (7), and CDPK4 (8). Several other proteins implicated in this process include a mitogen-activated protein kinase, MAP2 (9), and an ARID-domain containing protein, *Pf*ARID (10).

In most eukaryotes, typical cell cycle stages include cell growth (interphase), replication of its chromosomes (S phase) and cell divisions (M phase), and 2 gap phases called G1 and G2 flanking S phase. Cell cycle progression relies upon post-translational mechanisms including cell cycle kinases and phosphatases. The cyclin-dependent kinases (CDKs) are important signaling proteins regulating the cell cycle in various organisms (11, 12). CDK kinase activity is regulated by their interactions with cyclins and CDK inhibitors (CKIs) (12). Mammalian CDK1-4 and CDK6 regulate cell cycle progression, while CDK5 is involved in neuronal/synaptic functions, circadian clocks, DNA damage, cell cycle reentry, and mitochondrial dysfunction (13). Other CDKs such as CDK8-11 regulate gene expression and the cell cycle (14–16). The Cyclin proteins were initially discovered and named because their expression levels markedly fluctuate during the cell cycle. Cyclins control the kinase activity and substrate specificity of CDKs (16). The cell cycle in Plasmodium spp., deviates significantly from the typical eukaryotic cell cycle and produces hundreds of new daughter cells during a single replicative cycle. Plasmodium genomes encode 8 members of the CDK protein kinase family namely, CRK1 (PF3D7_0417800), CRK2/Protein kinase 5 (PF3D7_1356900), CRK3 (PF3D7_0415300), CRK4 (PF3D7_0317200), CRK5 (PF3D7_0615500), Protein kinase 6 (PF3D7_1337100), MO15-related protein kinase, MRK (PF3D7_1014400), an unannotated CDK kinase PF3D7_1338900 (17), and 3 cyclins Cyc1 (PF3D7_1463700), Cyc3 (PF3D7_0518400) and Cyc4 (PF3D7_1304700). The cyclin-dependence for *in vitro* kinase activity has been demonstrated for 2 of these, *Pf*PK5 (18) and *Pf*MRK (19), but their functional dependence in the parasite is not known.

During erythrocytic schizogony, nuclear division are asynchronous and independent often leading to formation of odd number of nuclei per schizont (20). *Pf*CRK4 is known to be an essential S phase regulatory factor required for initial and subsequent rounds of DNA replication (21). *Pf*CRK5 was reported to be important for the asexual proliferation and nuclear divisions of parasite (22). We have revisited the role of CRK5 in P. falciparum sexual stage development. We created *Pfcrk5^−^* parasites and in contrast to previous findings (22), *Pfcrk5^−^* parasites do not show a growth defect in asexual stages. We show that *PfCRK5* has a function in male gametogenesis and thus parasite transmission to the mosquito vector.

## RESULTS

### *Pf*CRK5 is expressed in the asexual and sexual stages of the parasite.

**FIG 1:**
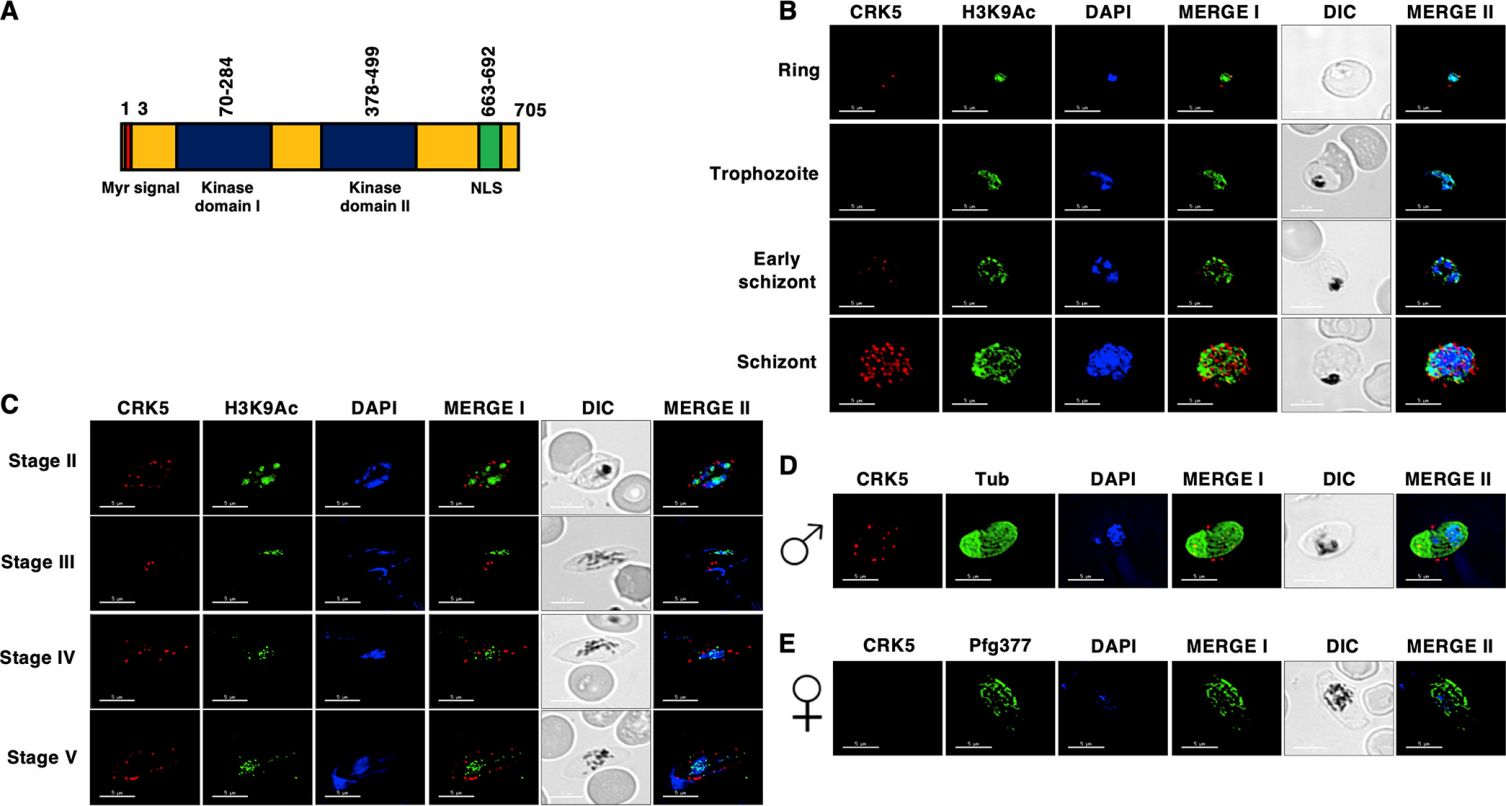
Expression and localization of *Pf*CRK5 in asexual and sexual stages. (A) Schematic for various motifs and domains of *Pf*CRK5 showing an N-terminal myristoylation signal (red) followed by 2 bipartite domains (blue) and nuclear localization signal (NLS) (in green). (B) Immunofluorescence assays were performed on WT NF54 asexual blood stages (ring, trophozoite and schizont) to colocalize *Pf*CRK5 (red) in combination with Histone marker H3K9Ac (green). The parasite nucleus was localized with 4′,6-diamidino-2-phenylindole (DAPI) (in blue). Scale bar = 5 μm. (C) Immunofluorescence assays were performed on WT NF54 sexual (stage II-V gametocytes) using thin culture smears and anti-*Pf*CRK5 antisera (in red) in combination with H3K9Ac (green). (D) and (E) Immunofluorescence assays were performed on stage V gametocytes using thin smears and anti-*Pf*CRK5 antisera (in red) either in combination with α-Tubulin (marker for male gametocytes, in green) or anti- *Pf*g377 (marker for female gametocytes, in green). Parasite nucleus was visualized with DAPI (blue). Scale bar = 5 μm.

The domain architecture of *Pf*CRK5 shows that it has N-terminal myristoylation signal, a single kinase domain with a spacer dividing it into 2 kinase subdomains, and a c-terminal nuclear localization signal (NLS) (Fig. 1A). To analyze the expression of *Pf*CRK5, a previously generated peptide antisera was used (22). Western blotting performed on WT and *Pfcrk^−^* parasites using anti-*Pf*CRK5 showed no *Pf*CRK5 signal in *Pfcrk5^−^* parasites, confirming the absence of *Pf*CRK5 protein and also the specificity of the antisera (Fig. S1A). Indirect immunofluorescence assays (IFAs) performed on thin blood smears of *in vitro* cultured *Pf*NF54 revealed that *Pf*CRK5 is expressed in the ring and schizont stages with a peri-nuclear localization (Fig. 1B), which is consistent with previous studies (22). *Pf*CRK5 expression was also detected in gametocytes from stage II through stage V in the cytoplasm, near-nucleus, and membrane (Fig. 1C). Dual fluorescence IFAs with male (anti-tubulin) or female (anti-*Pf*g377) gametocyte specific antibodies revealed that *Pf*CRK5 is expressed in male gametocytes (Fig. 1D and E), suggesting a male-specific function.

10.1128/mbio.02227-22.1 FIG S1 (A) Western blot analysis of *Pf*CRK5 in WT *Pf*NF54 and *Pfcrk5^−^* gametocytes, showing absence of *Pf*CRK5 in *Pfcrk5^−^* parasites. Actin abundance is shown as the loading control. (B) The Giemsa-stained thin culture smears were prepared for observing mature schizont stages for WT *Pf*NF54 and *Pfcrk5^−^* parasites at 1,000×magnifcation. Representative Giemsa-stained images of WT *Pf*NF54 and *Pfcrk5^−^* schizonts are shown. (C) The Giemsa-stained thin culture smears were used for quantitative assessment of number of daughter merozoites per schizont for WT *Pf*NF54 and *Pfcrk5^−^* parasites. (Duplicate experiments; *n* = 50 cells per experiment; bars are SD). NS, Not significant. Download FIG S1, TIF file, 0.9 MB. Copyright © 2022 Kumar et al. 2022 Kumar et al. https://creativecommons.org/licenses/by/4.0/ This content is distributed under the terms of the Creative Commons Attribution 4.0 International license.

### *PfCRK5* is not required for intra-erythrocytic parasite development.

**FIG 2:**
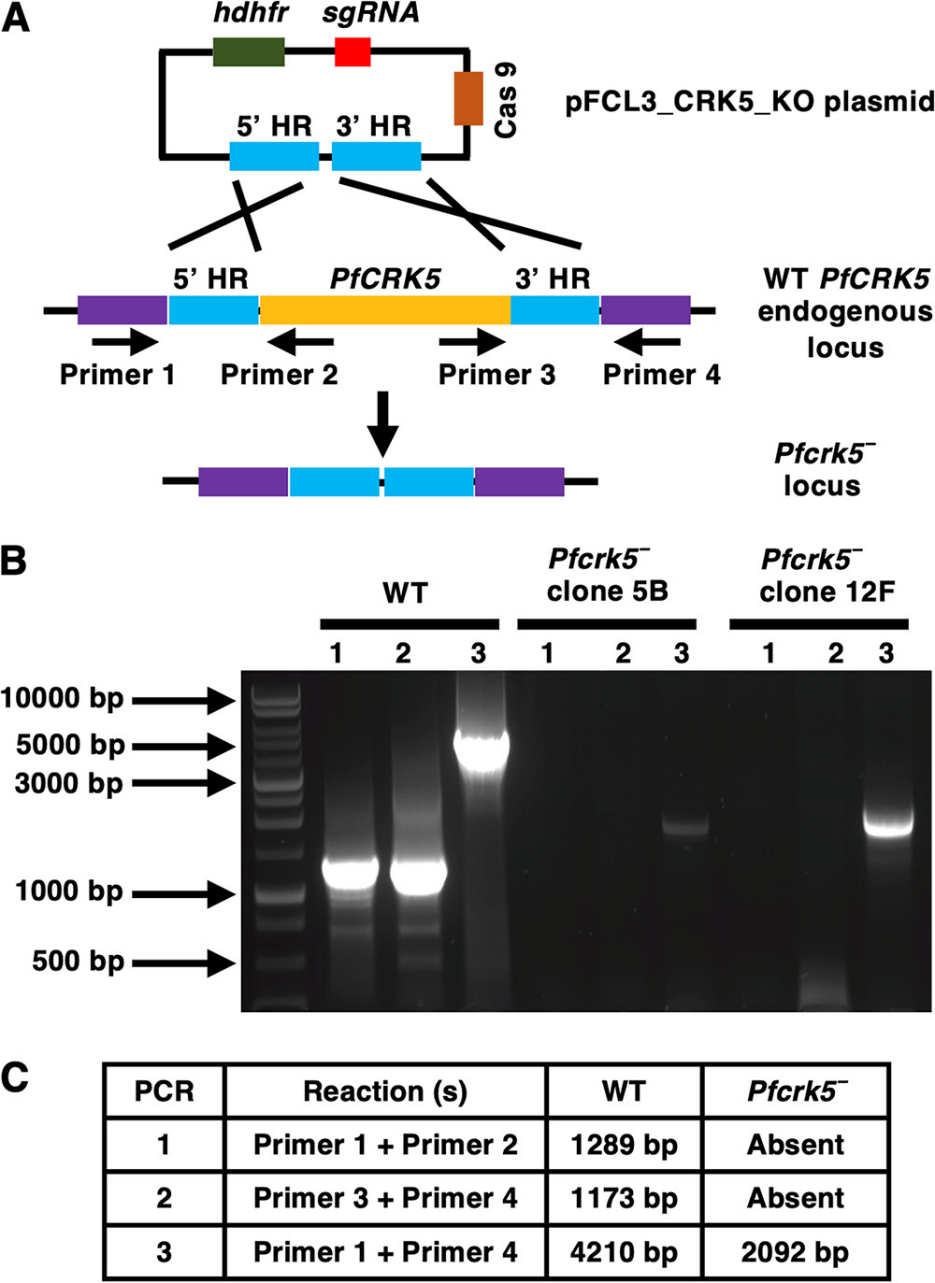
Generation of *Pfcrk5^−^* parasites. The schematic shows the strategy for deleting *PfCRK5*. The pFCL3_CRK5_KO plasmid has homology regions 5′ (5’HR) and 3′ (3’HR) not of the *PfCRK5* locus, a guide RNA sequence (sgRNA) and human dihydrofolate reductase (hDHFR) locus and Cas 9 cloned. The oligonucleotides were designed from outside 5’HR and 3’HR and *PfCRK5* locus and positions are indicated by arrows in (A). (B) Confirmation of *PfCRK5* deletion by diagnostic PCR. The expected sizes for different set of PCRs are indicated in (C).

**FIG 3:**
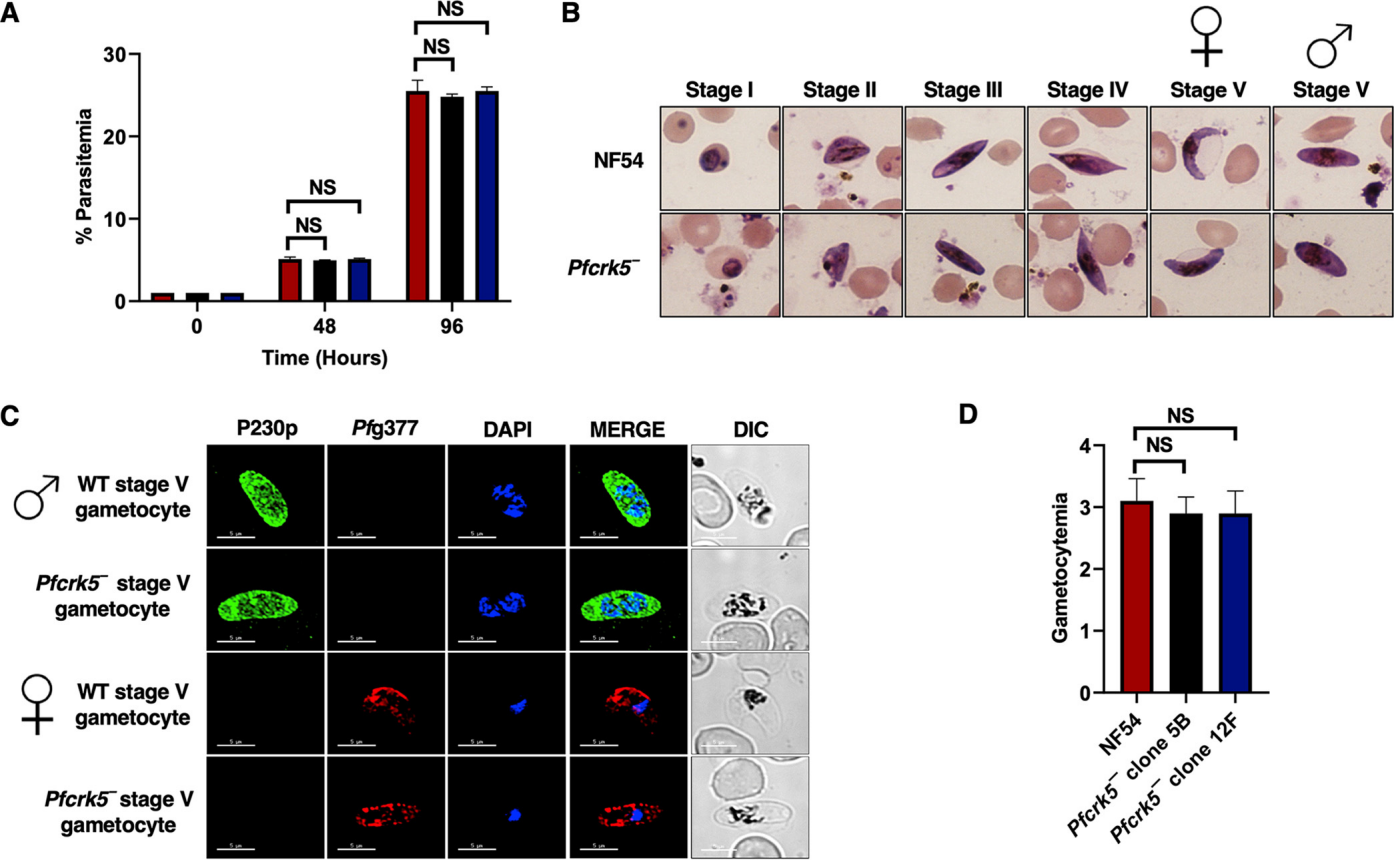
*Pfcrk5^−^* asexual stages grow normally and undergo gametocytogenesis. (A) Ring stage synchronous cultures for WT and two clones of *Pfcrk5^−^* (clone 5B and 12F) were plated to measure parasite growth over the course of 2 erythrocytic cycles. Total parasitemia was determined by counting the parasites from Giemsa-stained thin blood smears. Data were averaged from three biological replicates and presented as the mean ± standard deviation (SD). ns, not significant unpaired two-tailed Student's *t* test. (B) Ring stage synchronous cultures for WT and 2 different clones of *Pfcrk5^−^* (clone 5B and 12F) were tested for their potential to form gametocytes. Light microscopy of Giemsa-stained smears showing development of WT *Pf*NF54 and *Pfcrk5^−^* gametocytes and the 5 (I-V) distinct morphological stages. 1,000×magnifcation. Symbols for female and male gametocytes are shown on top of stage V gametocytes. (C) IFAs were performed on WT *Pf*NF54 and *Pfcrk5^−^* mature stage V gametocytes thin culture smears using anti-*Pf*P230p antisera, a marker for stage V male gametocytes (in green), in combination with anti-*Pf*g377 antisera, a marker for female gametocytes (in red). Representative images are shown. The parasite DNA was visualized with DAPI (blue). Scale bar = 5 μm. Merge I- merged image for red and green panels. Merge II- merged image for red, green, and DAPI (blue) channel. DIC, differential interference contrast. DAPI, 4′,6-diamidino-2-phenylindole. Symbols for male and female gametocytes are shown on left side of the image panels. (D) Gametocytemia was measured on day 15 using thin Giemsa-stained smears. Data were averaged from 3 biological replicates and presented as the mean ± standard deviation (SD). NS, Not significant.

For functional analysis, the endogenous *PfCRK5* gene was deleted using CRISPR/Cas9 (Fig. 2A). Gene deletion parasites (*Pfcrk5^−^*) were confirmed by a set of diagnostic PCRs with oligonucleotides specific for the *PfCRK5* locus and its upstream (5′) and downstream (3′) regions (Fig. 2A to C). Two individual clones for *Pfcrk5^−^* parasites (clone 5B and 12F) were used for phenotypic characterization. To analyze the role of *Pf*CRK5 in asexual parasite stages, a comparative growth assay was set up using *Pfcrk5^−^* parasites (clone 5B and 12F) along with wildtype (WT) NF54 parasites. Parasite growth was monitored over 2 asexual replication cycles. Giemsa-stained thin smears prepared every 48 h from the *in vitro* culture indicated that the growth rate of *Pfcrk5^−^* parasites was similar to WT NF54 parasites (Fig. 3A). We quantified the number of daughter merozoites per schizont for *Pfcrk5^−^* in comparison to WT NF54 parasites. This revealed that the average number of merozoites per schizont in *Pfcrk5^−^* was similar to WT *Pf*NF54 parasites (Fig. S1B and C).

### *Pfcrk5^−^* parasites undergo gametocytogenesis but fail to form microgametes.

**FIG 4:**
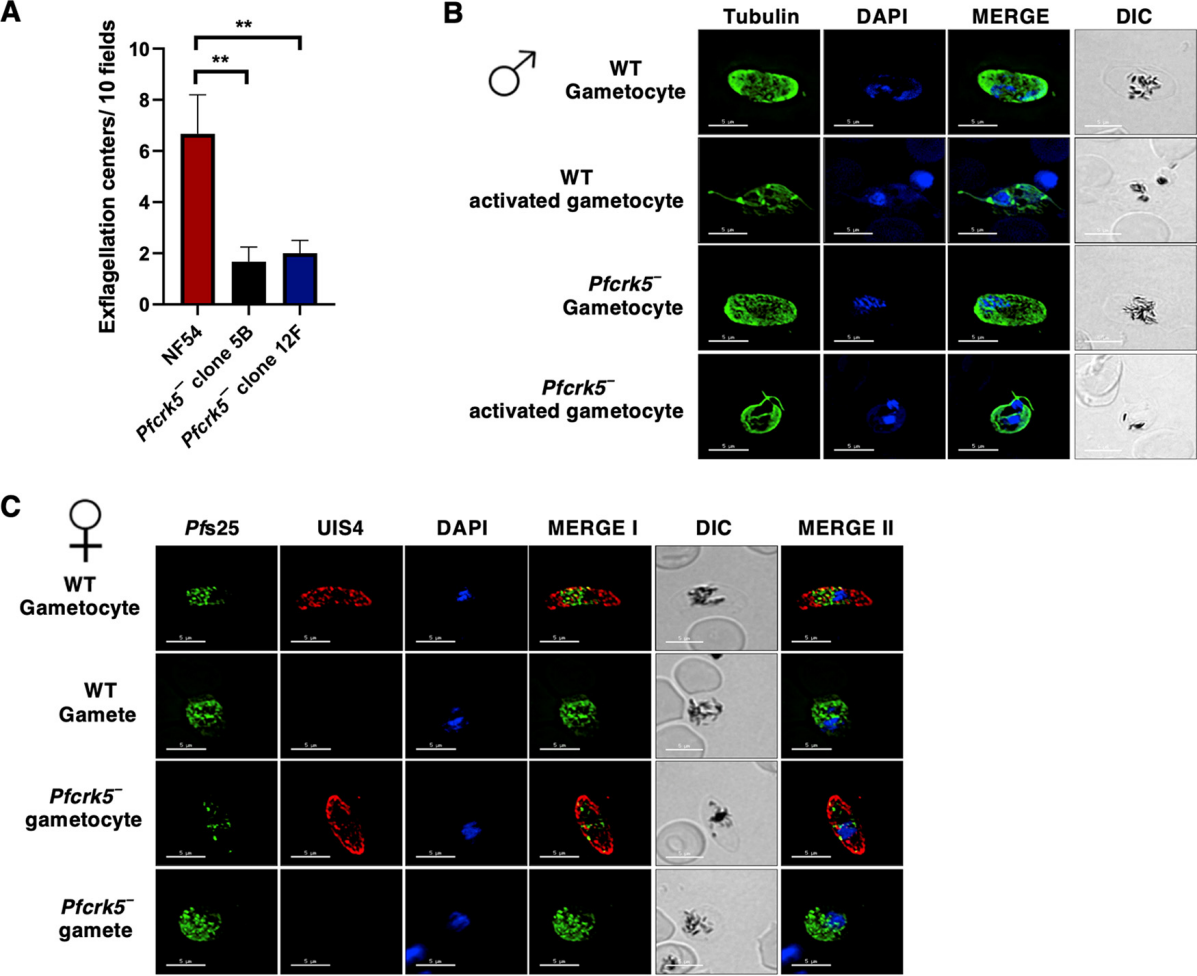
The *Pfcrk5^−^* parasites do not undergo male gametogenesis. (A) Number of exflagellation centers per field at 15 min post activation were enumerated. Data were averaged from 3 biological replicates and presented as the mean ± standard deviation (SD). (B) and (C) IFAs performed on thin blood smears of mature stage V gametocytes activated for 20 min *in vitro* for WT or *Pfcrk5^−^* (clone 12F) and were stained for α-tubulin (green), a male-specific marker, and *Pf*s25 (green), a marker for female gametes in an IFA. Anti-*Pf*UIS4 was used to stain parasitophorous vacuolar membrane. α-Tubulin staining showed male gametes emerging from an exflagellating male gametocyte in the WT parasite. The *Pfcrk5^−^* gametocytes were defective for male gametocyte exflagellation. Female gametes did not show any defect in egress from gametocyte body.

We next analyzed the ability of *Pfcrk5^−^* parasites to generate gametocytes. For this, *Pfcrk5^−^* parasites (clone 5B and 12F) along with WT NF54 parasites were used, and gametocytemia was scored for all cultures on day 15 of *in vitro* culture using Giemsa-stained culture smears and microscopic inspection. *Pfcrk5^−^* parasites were able to undergo gametocytogenesis, developing through stage I-V gametocytes, and could develop into mature stage V male and female gametocytes (Fig. 3B and C) with the gametocytemia being similar to WT NF54 (Fig. 3D). We next analyzed whether *Pfcrk5^−^* gametocytes undergo gametogenesis. Day 15 gametocyte cultures for WT NF54 and *Pfcrk5^−^* were activated by addition of O^+^ human serum and dropping the temperature from 37°C to room temperature (RT). Activated gametocytes were used to prepare a temporary live, wet mount of cultures and exflagellation centers were measured in 15 random fields of microscopic view at ×40 magnification. Strikingly, the number of exflagellation centers for *Pfcrk5^−^* (Fig. 4A) were significantly reduced, indicating an exflagellation defect. To confirm this defect, IFAs were performed with thin culture smears for WT NF54 and *Pfcrk5^−^* activated gametocytes 15 min post activation, and parasites were stained with an anti-tubulin antibody. The lack of release of observable male gamete exflagella from the gametocyte body confirmed an exflagellation defect in *Pfcrk5^−^* (Fig. 4B). Female *Pfcrk5^−^* gametes were stained with *Pf*s25 antibody and UIS4 antibody, which marks parasitophorous vacuole membranes. No observable defect was seen in *Pfcrk5^−^* (Fig. 4C). These results indicate *Pf*CRK5 is critical for male gametogenesis.

### The *Pfcrk5^−^* male defect causes a severe reduction in transmission to the mosquito vector.

**FIG 5:**
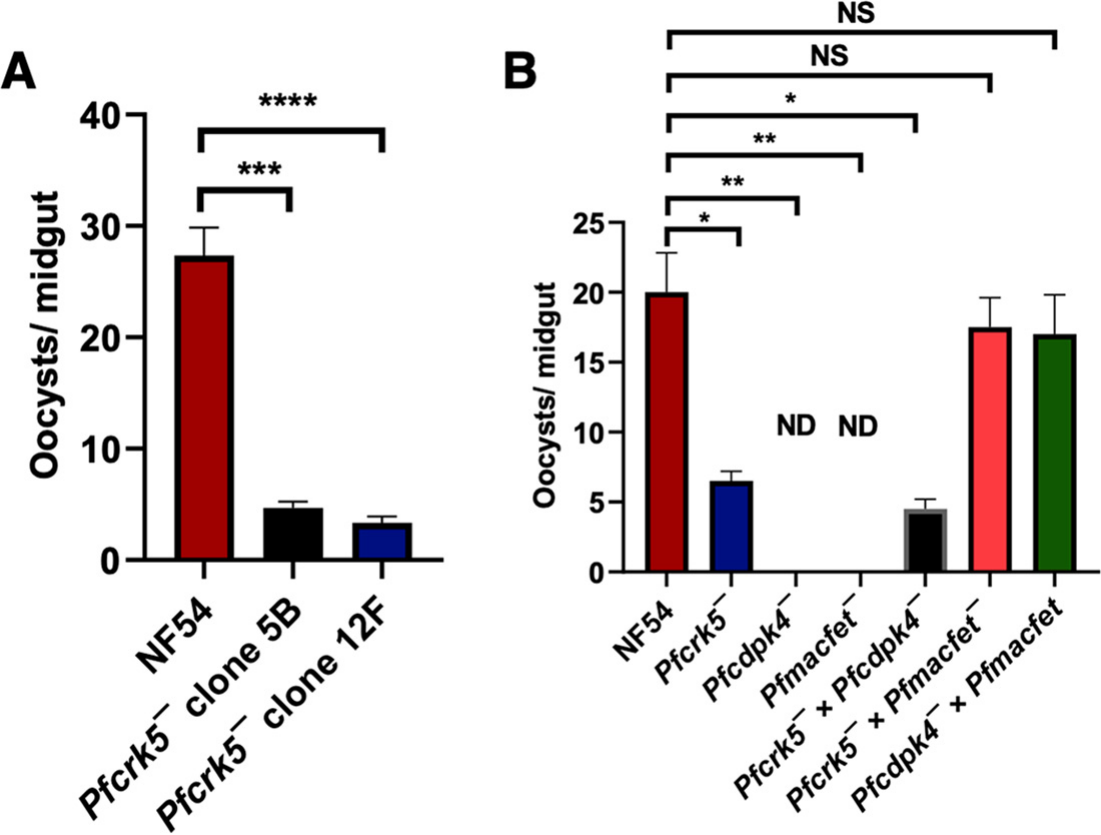
The *Pfcrk5^−^* parasites show robust reduction in infection in mosquitoes. (A) A. stephensi mosquitoes were dissected on day 7 post feed and number of oocysts were enumerated per midgut. Data were averaged from 3 biological replicates with a minimum of 50 mosquito guts and presented as the mean ± standard deviation (SD). (B) A. stephensi mosquitoes were dissected on day 7 post feed and number of oocysts were enumerated per midgut for WT *Pf*NF54, *Pfcrk5^−^*, *Pfcdpk4^−^*, *Pfmacfet^−^*, *Pfcrk5^−^* × *Pfcdpk4^−^*, *Pfcrk5^−^* × *Pfmacfet^−^*, *Pfcdpk4^−^* × *Pfmacfet^−^*. *In vitro* genetic crosses revealed that the *Pfcrk5^−^* showed productive cross-fertilization with the female sterile *Pfmacfet^−^* parasites, while it was strongly reduced with the male sterile *Pfcdpk4^−^* parasites (error bar indicates mean ± SD; *n* = 2). ND, Not detected. NS, Not significant.

We next examined the transmissibility of *Pfcrk5^−^* gametocytes to female Anopheles stephensi mosquitoes. Infectious blood meals of WT and *Pfcrk5^−^* stage V gametocytes were prepared using standard methods and fed to mosquitoes via membrane feeders. Mosquito midguts were dissected on Day 7 post feed, which revealed that *Pfcrk5^−^* parasites displayed a severe reduction in number of oocysts in comparison to well-infected WT controls (Fig. 5A). These results revealed that *Pf*CRK5 is important for transmission to the mosquito vector via a crucial function in male gametogenesis. After determining the role of *Pf*CRK5 in male gametocyte exflagellation, we sought to determine the fertility of individual sex. Since it is not possible to separate male and female gametocytes in *in vitro* culture, we further analyzed the fertility of male and female *Pfcrk5^−^*gametes utilizing genetic crosses as described previously (10). For this, we used *Pfcrk5^−^* parasites and transgenic parasite lines, which are sex-sterile for one sex forming either fertile female gametes only (*Pfcdpk4^−^*) (8) or fertile male gametes (*Pfmacfet^−^*) only (23), as described previously (10). WT NF54, *Pfcrk5^−^*, *Pfcdpk4^−^*, and *Pfmacfet^−^* gametocytes were generated *in vitro* in culture for 15 days, and cultures were first fed individually to mosquitoes. For crosses, the gametocytes from these parasites were mixed in equal ratio as follows: *Pfcrk5^−^* × *Pfcdpk4^−^*, *Pfcrk5^−^* × *Pfmacfet^−^*, *Pfcdpk4^−^* × *Pfmacfet^−^*. Mosquitoes were dissected on day 7 post feeding to enumerate midgut oocysts for all the feeds. While WT *Pf*NF54 gametocytes infected mosquito midguts robustly, *Pfcrk5^−^* gametocytes showed a strong reduction in oocyst numbers (Fig. 5B), and the *Pfcdpk4^−^* and *Pfmacfet^−^* did not show any infection as expected. The *Pfcrk5^−^* × *Pfcdpk4^−^* cross showed highly reduced number of oocysts. However, in the *Pfcrk5^−^* × *Pfmacfet^−^* cross, oocysts were observed. This indicated productive fertilization of *Pfcrk5^−^* female gametes by *Pfmacfet^−^* male gametes (Fig. 5B). Oocyst development was also observed in *Pfcdpk4^−^* × *Pfmacfet^−^* (positive control) cross. These experiments demonstrate that *Pf*CRK5 is important for male gametogenesis.

## DISCUSSION

The uptake of gametocytes by *Anopheline* mosquitoes is critical for the completion of the sexual phase of the parasite life cycle. Upon encountering cellular triggers in the mosquito midgut, gametocytes rapidly form gametes that move through the blood meal, undergo fertilization to form zygotes, followed by differentiation into other mosquito stages. However, proteins that are critical for the formation of fertilization-competent gametes and sexual reproduction, particularly for human malaria parasites, are unknown. Our study demonstrates that *Pf*CRK5 is important for male gametogenesis and transmission to the mosquito.

Signaling proteins such as kinases are key regulators across various life cycle stages of the malaria parasite (24–27). Protein kinases such as *Pf*CDPK1 (6), *Pf*CDPK2 (7), and *Pf*CDPK4 (8) are involved in gametogenesis and are critical for establishing infection of the mosquito vector. Other kinases such as *Pf*PKG (28), *Pf*MAP2 (9), and *Pf*SRPK1 (Kumar et al., 2022; DOI: 10.1128/spectrum.02141-22) play a role in gametogenesis, indicating the importance of phospho-signaling events in sexual development of the parasite. Other proteins regulating gametogenesis in *Pf* include *Pf*g377 (29), M-TRAP (merozoite-thrombospondin-related anonymous protein) (30) and perforin-like protein (PPLP2) (31).

In this study, we show that *Pf*CRK5 is expressed throughout asexual blood stage development and gametocyte development. *Pf*CRK5 displays a peri-nuclear localization in asexual stages, while in sexual stages it shows more cytoplasmic or membrane localization. We further demonstrate that *Pf*CRK5 exhibits a male gametocyte specific expression which can be relevant to its cellular function. *Pf*CRK5 possesses an N-terminal myristoylation signal and a c-terminal nuclear localization signal. The myristoylation signal for various kinases has been shown to regulate their membranous localization in plants (32) and Plasmodium spp. (33). Therefore, both myristoylation signal and nuclear localization signals may be regulating *Pf*CRK5 cellular localization in various stages which may be relevant to its function.

A previous report with *Pfcrk5^−^* parasites has shown that *Pfcrk5^−^* parasites show a growth defect in asexual blood stages due to a defective number of daughter merozoites per schizont (22). However, in our experiments, we did not observe any growth defect in *Pfcrk5^−^* parasites. It is possible that the growth defect may arise due to long-term parasite culture and/or culture conditions. Another study on the rodent malaria parasite Plasmodium berghei *Pb*CRK5 have also shown that the gene deletion parasites do not exhibit any growth defect (34). Our experiments show that even if *Pf*CRK5 is expressed in the asexual blood stage and throughout gametocyte development, it is not required for asexual blood stage proliferation or gametocyte development. There is a possibility of the compensation of CRK5 kinase activity during asexual schizogony by other kinases such as CRK4, since it is also important for DNA replication during this developmental stage (21). As a result, deletion of *PfCRK5* is not lethal for asexual development which involves DNA replication. This hypothesis is based upon a previous study that demonstrates compensation of *Pf*CDPK1 kinase activity in asexual stages through the action of another protein kinase *Pf*PKG (35).

We found that *Pfcrk5^−^* parasites develop into mature stage V male and female gametocytes. While female *Pfcrk5^−^* gametocytes undergo gametogenesis, male gametocytes undergo activation and form spheroid cells but exhibit a severe defect in male gametogenesis. Mosquito feeding experiments revealed that *Pfcrk5^−^* parasites show a robust defect in transmission which can be attributed to a defect in male gametogenesis. Further genetic crosses experiments with sex-sterile transgenic parasite lines revealed that *Pfcrk5^−^* female gametes are fertile but male gametes suffer severe defects in fertility.

In eukaryotic cells, cell cycle progression is regulated by interplay between cyclins and cyclin-dependent protein kinases (CDKs), along with additional protein complexes such as the anaphase promoting complex (APC), which regulates cyclin degradation (36, 37). While still inside the erythrocytes, male gametocytes (which are in the G1 phase of the cell cycle) undergo 3 rapid rounds of DNA replication and start assembling the flagellum (38). During male gametogenesis, there is no detectable karyokinesis and cytokinesis is uncoupled from DNA replication, indicating a lack of cell cycle checkpoints (38). In fact, Plasmodium cyclins do not show oscillating expression profiles, though stable complexes of cyclins and CRKs have been reported (39), which may represent a parasite specific cell cycle machinery. Two of the four Plasmodium cyclins (*Pf*Cyc1 and *Pf*Cyc4) are known to interact with *Pf*CRK5 *in vitro* and regulate its activity (22), suggesting a cyclin-dependence for *Pf*CRK5 although coimmunoprecipitation of *Pf*Cyc1 from parasite lysates identified *Pf*MAT1 and *Pf*MRK as specific interactors (39). This suggests that *Pf*CRK5 may interact with *Pf*Cyc4 or some other cyclin like protein in the parasite in a stage specific manner. In the rodent malaria parasite P. berghei, *Pb*CRK5 is known to interact with a predicted parasite cyclin SOC2, although there is no evidence of SOC2 cycling by transcription, translation, or degradation (34). *Pb*CRK5 phosphorylates components of pre-replicative complexes, such as proteins important in DNA replication (i.e., a licensing factor like protein chromatin licensing and DNA replication factor 1 [CDT1]), a possible orthologue of the DNA replication factor CDC6, and 2 ORC components (ORC2 and ORC4) (34). Previous studies have also indicated the role of *Pf*CDPK4 in male gametogenesis possibly by phosphorylation of *Pf*CDPK1, *Pf*SOC3, *Pf*SOC7 (a ribonucleoside-diphosphate reductase), and ATP-dependent6-phosphofructokinase (PFK9), replication components, such as replication factor C subunit 1, replication factor C subunit 4, DNA replication licensing factor CDT1, MCM4, DNA polymerase alpha catalytic subunit A, SAS6, and microtubule proteins Kinesin 13 and Kinesin 8B (8). This suggests that *Pf*CRK5 and *Pf*CDPK4 may target similar parasite proteins during gametogenesis. Therefore, it is reasonable to propose here that there may be a possible cross talk between CDPK4 and CRK5 which may be regulating phosphorylation of these key parasite proteins. These mechanisms may be responsible for the role of *Pf*CRK5 in regulating DNA replication and exflagella formation during male gametogenesis.

In conclusion, our study shows that *Pf*CRK5 plays a key role in male gametogenesis and transmission. Further studies are warranted to identify the molecular mechanisms via which it regulates male gametogenesis. Since *Pf*CRK5 is significantly divergent from mammalian CDKs, it could be an attractive target for developing kinase inhibitors that block malaria transmission.

## MATERIALS AND METHODS

### Reagents and primary antibodies.

All molecular biology reagents were purchased from Millipore Sigma, unless otherwise stated. All oligonucleotides were purchased from IDT Inc. The following primary antibodies/antisera and dilutions were used: mouse anti-tubulin antibody (1:200, Millipore Sigma, cat# T5168); mouse anti-*Pf*P230p (1:200, kindly gifted by Professor Kim C. Williamson, Uniformed Services University of the Health Sciences, USA) (40), mouse anti-*Pf*g377 (1:250, kindly gifted by Professor Pietro Alano at Istituto Superiore di Sanità, Italy), mouse anti-H3K9Ac (1:100, MABI0305, GeneTex), and *Pf*CRK5 (1:50, kindly provided by Professor Christian Doerig, RMIT university, Australia) (22). All Alexa fluor conjugated secondary antibodies were purchased from ThermoFisher Scientific.

### P. falciparum culture and transfection.

The P. falciparum NF54 and *Pfcrk5^−^* parasites, asexual and sexual cultures were maintained as described elsewhere (10). The oligonucleotides used for creation and genotyping analysis of *Pfcrk5^−^* parasites are detailed in Table S1. Deletion of *PfCRK5* (PlasmoDB identifier Gene - PF3D7_0615500) was achieved as described elsewhere (10). Two individual clones for *Pfcrk5^−^* (clone 5B and 12F) were used for phenotypic analysis.

10.1128/mbio.02227-22.2 TABLE S1 Oligonucleotides used in the study. Download Table S1, DOCX file, 0.02 MB. Copyright © 2022 Kumar et al. 2022 Kumar et al. https://creativecommons.org/licenses/by/4.0/ This content is distributed under the terms of the Creative Commons Attribution 4.0 International license.

### Growth assays and measurement of gametocyte development.

The comparative growth for asexual blood stage between the *Pf*NF54 WT and *Pfcrk5^−^* parasites was assessed as described elsewhere (10). To compare gametocyte formation between WT *Pf*NF54 and *Pfcrk5^−^*, gametocytes were cultured as described elsewhere (41), and gametocytemia was enumerated using Giemsa-stained thin culture smears on day 15 of *in vitro* culture.

### Exflagellation, standard membrane feeding assay, and oocyst measurements.

The assessment of comparative exflagellation, standard membrane feeding assay (SMFA), and oocyst measurements were performed as described elsewhere (10).

### Indirect immunofluorescence.

IFAs were performed on asexual and sexual blood stage parasites and exflagellating microgametocytes using thin smears prepared on Teflon coated slides as described elsewhere (42). Antigens were visualized using anti-species antibodies. Images were acquired using a 100 × 1.4 NA objective 90 (Olympus) on a Delta Vision Elite High-Resolution Microscope (GE Healthcare Life Sciences).

### Statistical analysis.

All data related to phenotyping assays are expressed as mean ± SD. Statistical differences were deemed significant using *P*-values from an unpaired, two-tailed Student's *t* test. Values of *P* < 0.05 were considered statistically significant. Significances were calculated using GraphPad Prism and are represented in the Figures as follows: ns, not significant, *P* > 0.05; *, *P* < 0.05; **, *P* < 0.01; ***, *P* < 0.001.

### Data availability.

All other relevant data are available from the authors upon reasonable request.
