# Supplementary figures and images for: Plasmodium falciparum CRK5 Is Critical for Male Gametogenesis and Infection of the Mosquito
Source: mBio. 2022 Sep 26;13(5):e02227-22. doi: 10.1128/mbio.02227-22 (PMC9600428; doi:10.1128/mbio.02227-22)

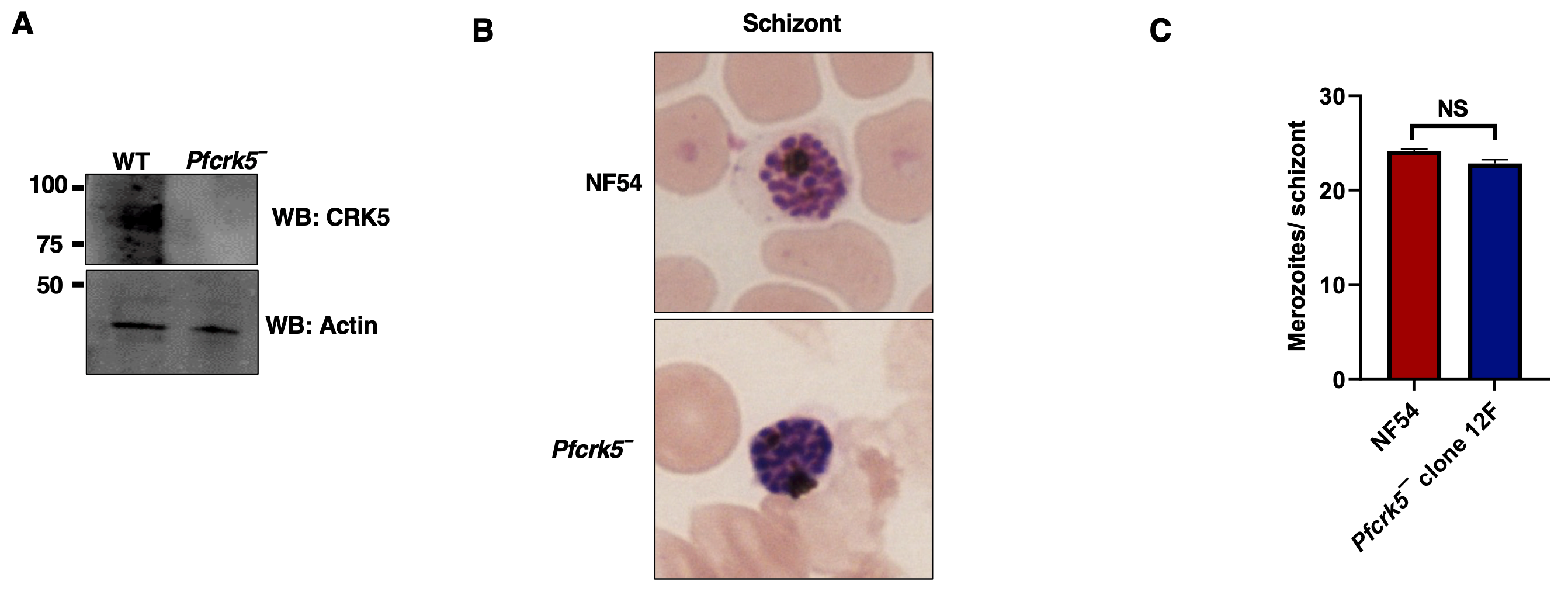

Supplement: FIG S1 [file mbio.02227-22-s0001.tif]
