# Supplementary material for: Plasmodium falciparum CRK5 Is Critical for Male Gametogenesis and Infection of the Mosquito
Source: mBio. 2022 Sep 26;13(5):e02227-22. doi: 10.1128/mbio.02227-22 (PMC9600428; doi:10.1128/mbio.02227-22)
Supplement: TABLE S1 [file mbio.02227-22-s0002.docx]

**Supplementary Table S1: Oligonucleotides used in the study.**

| **Oligonucleotides used for generation of *Pfcrk5¯* parasites** | |
| --- | --- |
| **Oligo** | **Forward (5’-3’)** |
| *PfCRK5* 5’Homo For | T**GCGGCCGC**GTACAAACCAGTACATTTTGTACTAATGTATATAAATTATTGT |
| *PfCRK5* 5’Homo Rev | CCAACCCGGGTATAGGCGCGCCTGTGATATGAAACGAAACGATATGAAATAAAACGATAT |
| *PfCRK5* 3’Homo For | AGGCGCGCCTATACCCGGGTTGGGTATAATTGGATGTAAACAAAACATTTTTGTTACAAT |
| *PfCRK5* 3’Homo Rev | TAA**GTCGAC**GGCGTATATTCACAATTCTATTAAAAGTATGTAC |
| *PfCRK5* Guide 1 For | **TATT**ATCTCGAAGAAAAATTAGGGG |
| *PfCRK5* Guide 1 Rev | **AAAC**CCCCTAATTTTTCTTCGAGAT |
| *PfCRK5* Guide 2 For | **TATT**AGGTGTTGAAAAATCTGGAA |
| *PfCRK5* Guide 2 Rev | **AAAC**TTCCAGATTTTTCAACACCT |
| *PfCRK5* Geno5 For | GTCATAGCAATTTCGTTTTTTTAAATAAGAGATG |
| *PfCRK5* Geno5 Rev | CTTAAATCGTCTCGAAAAAATTTTATGGC |
| *PfCRK5* Geno3 For | GAAATCTATCCATTCCAGATTTTTCAACA |
| *PfCRK5* Geno3 Rev | CTCATTTTATTATAAGTGATAAGATAGAATCGTTAGGT |
